# Twice or thrice weekly levothyroxine provides similar rates of adherence and post-Ramadan euthyroidism compared to daily levothyroxine during Ramadan fasting

**DOI:** 10.1186/s13044-023-00187-z

**Published:** 2023-12-06

**Authors:** Tamer Mohamed Elsherbiny

**Affiliations:** Endocrine division – Alexandria faculty of medicine, Khartoum square, Azarita, Alexandria Egypt

**Keywords:** Ramadan, Levothyroxine, Fasting, Weekly, Hypothyroidism.

## Abstract

**Background:**

Having to take levothyroxine (L-T4) on a daily basis, on an empty stomach is burdensome and may impair adherence, especially during Ramadan fasting. A long half-life and autoregulation of thyroid hormone levels allows for twice or thrice weekly administration of L-T4. This study aims to establish twice or thrice weekly L-T4 dosing as a convenient and effective practice during Ramadan fasting.

**Methods:**

The study included 2 groups; twice or thrice weekly (TTW) group included patients assigned to take L-T4 twice or thrice a week, and standard daily dosing (SDT) group included patients assigned to take L-T4 daily. Patients freely chose between three L-T4 regimens: before Iftar, before Suhor, or before the next fast. Thyroid status was assessed before and within 6 weeks after Ramadan. Only euthyroid patients were included.

**Results:**

TTW group included 57 patients, while SDT group included 91 patients. Pre-Ramadan TSH in TTW group (1.80 ± 0.88 µIU/L) was higher compared to SDT group (1.39 ± 0.72 µIU/L) [P = 0.003]. Similar adherence rates were observed in both groups, 96.5% in TTW group versus 89% in SDT group, [P = 0.13]. similar rates of post-Ramadan euthyroidism were also found in both groups, 91.2% in TTW group versus 94.5% in SDT group, [P = 0.509]. TTW group preferred regimen 1 (64.9%) significantly more than SDT group (35.2%) [P = 0.001].

**Conclusion:**

Twice or thrice weekly levothyroxine results in similarly high rates of adherence (96.5%) and post-Ramadan euthyroidism (91.2%) compared to daily levothyroxine during Ramadan fasting.

## Background

Daily Levothyroxine (L-T4) is the standard treatment for hypothyroidism. For optimal absorption, it has to be taken on an empty stomach, in the early morning one hour before food and beverages or before sleep several hours after the last meal [1]. Achieving such conditions on a daily basis isn’t always feasible, leading to a high rate of non-adherence and dysthyroidism among hypothyroid patients [2].

Levothyroxine has an exceptionally long half-life of 7 days. Once weekly L-T4 has shown to be effective and safe in the management of treatment refractory hypothyroidism due to non- adherence [3]. Twice weekly and every other day L-T4 have also shown similar safety and efficacy compared to standard daily L-T4 [4, 5].

Adherence to L-T4 in Ramadan is complicated by the fact that only few hours are available to take levothyroxine in optimal conditions. In Ramadan, L-T4 may be taken one hour before Iftar meal, however, family gatherings at Iftar meal are frequent during Ramadan which makes this regimen unsuitable for some hypothyroid patients. An alternative regimen is to take it several hours after Iftar and one hour before Suhor meal allowing the patient to actively share in the family gatherings at Iftar meal, however, some patients may consider this a second nighttime fast in addition to the longer day time fast [6].

A small pilot study has been conducted as a proof of concept has shown safety and efficacy of twice or thrice L-T4 during Ramadan comparable to standard daily treatment [7]. The present study aims to confirm the results of the pilot study to establish twice or thrice weekly L-T4 as a safe, effective, and more convenient alternative to standard daily L-T4 during Ramadan.

### Subjects and methods

This was a prospective study including Muslim hypothyroid patients willing to fast Ramadan during the year 2021 attending endocrinology outpatient clinic, Alexandria faculty of medicine, Alexandria university, Egypt. All included patients were euthyroid, and stable on the same L-T4 dose for at least 3 months before the start of Ramadan. Exclusion criteria were thyroid cancer patients requiring suppressive therapy, central hypothyroidism, pregnancy, coronary heart disease, arrythmia, chronic heart failure, liver cirrhosis, renal failure, acute medical or surgical illness at the time of evaluation to avoid acute and chronic non thyroidal illness syndromes. All patients were explained the nature and aim of the study and signed an informed written consent. The protocol of the study was approved by the ethical committee of Alexandria faculty of medicine, [IRB number 12,098].

Recruited patients were divided into two groups. Twice or thrice weekly L-T4 group (TTW) included patients who took L-T4 on a daily basis before Ramadan and were switched at the beginning of Ramadan from standard daily L-T4 to twice or thrice weekly dosing. Their weekly dose – daily dose multiplied by 7 days – was divided equally into 2 or 3 doses, given at 2 or 3 successive fixed days of the week, Saturdays, Sundays, and Mondays. These days were chosen specifically to avoid family gatherings usually taking place at weekends, Thursdays, and Fridays. Patients reverted to daily L-T4 at the start of the first week after Ramadan.

Standard daily therapy Group (SDT) included patients who continued to take L-T4 on standard daily basis. Patients from both groups were free to follow one of three L-T4 regimens during Ramadan, explained in detail previously [8]. In short, regimen 1: to take L-T4 at sunset, 60 min before Iftar and beverages, regimen 2: to take L-T4 3–4 h after Iftar, 60 min before Suhor meal, regimen 3: to take L-T4 before the start of next fast 3–4 h after an early Suhor at midnight. If patients mixed between regimens 1 and 2, this was labeled regimen 4.

Adherence was assessed by interviewing participants during post-Ramadan visit. Non-adherence was defined as stopping food and beverages for less than 3 h before L-T4 tablet(s) or stopping food and beverages for less than 45 min after L- T4 tablet(s). Patients who skipped L-T4 treatment for 2 or more days without making up for their missed doses were excluded from the study.

Thyroid status was assessed for recruited patients in pre-Ramadan visit using TSH. The institution uses electrochemiluminescence immunoassay [ECLIA] on Cobas e 411 (Roche Diagnostics GmbH, Mannheim, Germany). Patients were considered euthyroid when TSH was 0.3-4 µIU/L for patients less than 70 years of age, and 1–5 µIU/L for patients more than 70 years of age according to European thyroid association (ETA) recommendations [9].

Thyroid status was reassessed in post-Ramadan visit using TSH, provided that this visit comes within 6 weeks from the end of Ramadan. Patients were excluded from the study if post-Ramadan visit was delayed beyond 6 weeks after Ramadan or if they did not report TSH during post-Ramadan visit.

### Statistical methods

Data was fed to the computer and analyzed using IBM SPSS software package version 20.0. **(**Armonk, NY: IBM Corp**).** The Kolmogorov- Smirnov was used to verify the normality of distribution of variables, Comparisons between groups for categorical variables were assessed using Chi-square test (Fisher or Monte Carlo). Mann Whitney test was used to compare between two groups for not normally distributed quantitative variables. Kruskal Wallis test was used to compare different groups for abnormally distributed quantitative variables and followed by Post Hoc test (Dunn’s for multiple comparisons test) for pairwise comparison. Spearman coefficient was used to correlate between quantitative variables. The significance of the obtained results was judged at the 5% level.

## Results

**Fig. 1:**
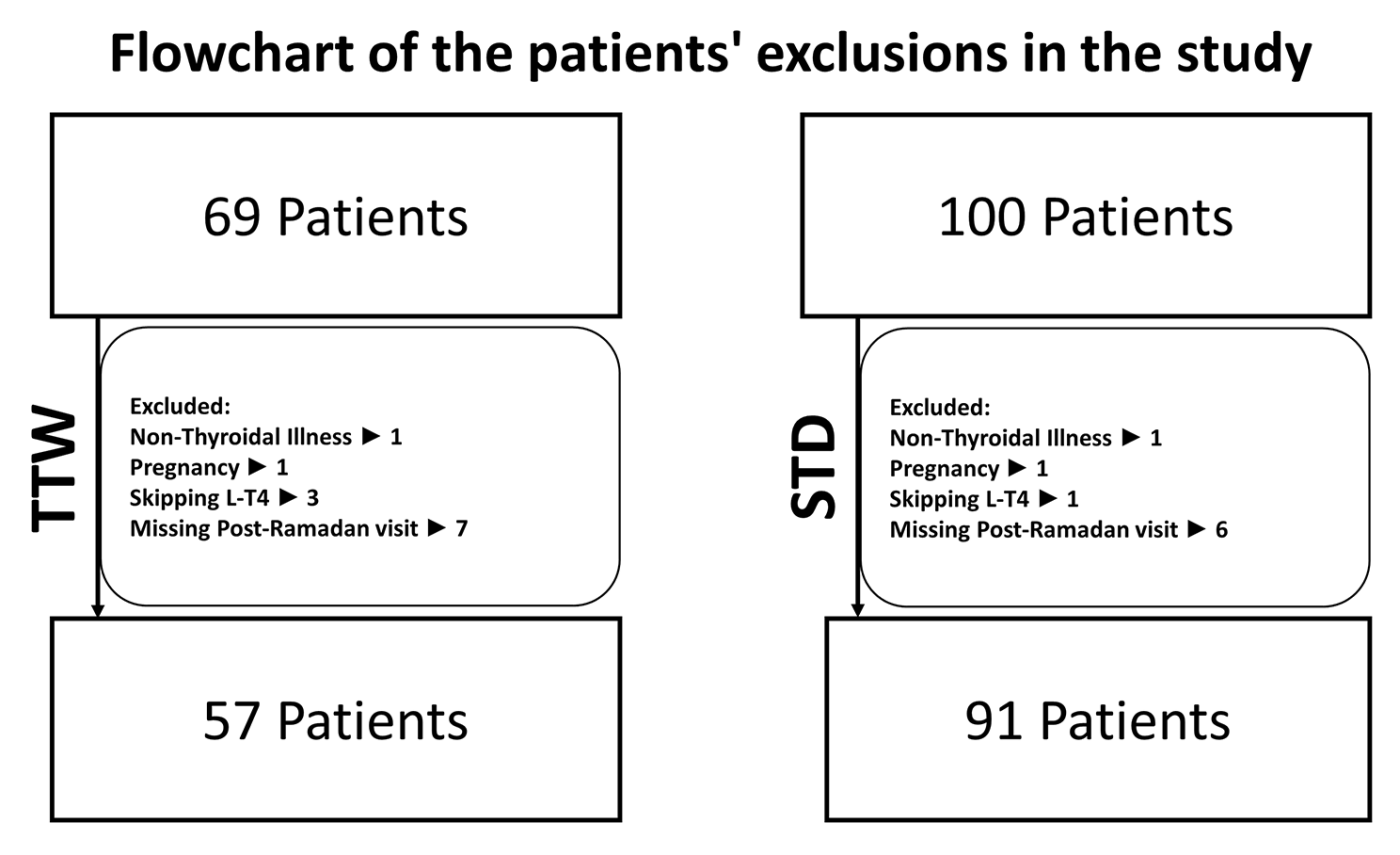
Flow chart of patient’s exclusions in the study. TTW: Twice or thrice weekly levothyroxine group, SDT: Standard daily therapy group

Twice or thrice weekly L-T4 group (TTW) included 57 patients, 13 (22.8%) patients received twice weekly L-T4, and 44 (77.2%) patients received thrice weekly L-4. Standard daily therapy Group (SDT) included 91 patients (Fig. 1).

### Baseline characteristics

Most patients in both groups were females, 98.2% of TTW group, and 92.3% of SDT group, [P = 0.153]. The mean and median levothyroxine doses were 90.6 and 85 µg/day for TTW group, and 107.2 and 100 µg/day for SDT group. Age was significantly lower in TTW group compared to SDT group, mean age in TTW group was 37.32 ± 11.01 years versus 46.97 ± 13.85 years in SDT group, [p < 0.001]. All included patients in both groups were euthyroid at inclusion. Pre-Ramadan TSH was significantly higher in TTW group compared to SDT group, mean TSH in TTW group was 1.80 ± 0.88 µIU/L versus 1.39 ± 0.72 µIU/L in SDT group [P = 0.003] (Table 1).

**Table 1:** Baseline data and post-Ramadan outcomes for the 2 studied groups

	TTW group (n = 57)	SDT group (n = 91)	Test of sig.	p
**Sex**				
Female	56 (98.2%)	84 (92.3%)	χ^2^ = 2.417	^FE^p= 0.153
Male	1 (1.8%)	7 (7.7%)		
**Age (years)**				
Mean ± SD.	37.32 ± 11.01	46.97 ± 13.85	t = 4.691^*^	< 0.001^*^
Median (Min. – Max.)	37 (18–60)	45 (17–78)		
**Diagnosis**				
HT	44 (77.2%)	62 (68.1%)	χ^2^= 6.901	^FE^p= 0.084
Tx	6 (10.5%)	23 (25.3%)		
RAI	5 (8.8%)	5 (5.5%)		
PPT	1 (1.8%)	1 (1.1%)		
Unclassified	1 (1.8%)	0 (0%)		
**Regimen number**				
1	37 (64.9%)	32 (35.2%)	χ^2^= 16.689^*^	^MC^p= 0.001^*^
2	17 (29.8%)	41 (45.1%)		
3	3 (5.3%)	7 (7.7%)		
4	0 (0%)	11(12.1)		
**Adherence**				
Adherent	55 (96.5%)	81 (89%)	χ^2^ = 2.632	^FE^p= 0.130
Non-adherent	2 (3.5%)	10 (11%)		
**Pre-Ramadan TSH**				
Mean ± SD.	1.80 ± 0.88	1.39 ± 0.72	U = 1850.5^*^	0.003^*^
Median (Min. – Max.)	1.81 (0.30–3.90)	1.17 (0.30–3.68)		
**Post-Ramadan TSH**				
Mean ± SD.	2.30 ± 1.25	1.69 ± 1.70	U = 1653.5^*^	< 0.001^*^
Median (Min. – Max.)	2.08 (0.08–7.50)	1.52 (0.08–15.1)		
**Post-Ramadan Thyroid status**				
Euthyroid	52 (91.2%)	86 (94.5%)	χ^2^ = 0.598	^FE^p= 0.509
Dysthyroid	5 (8.8%)	5 (5.5%)		

SD: Standard deviation U: Mann Whitney test t: Student t test

χ^2^: Chi square test FE: Fisher Exact

p: p value for comparing between the studied groups

*: significant at P value of < 0.05

TTW Twice or thrice weekly, SDT Standard daily therapy

HT Hashimoto thyroiditis, Tx thyroidectomy, RAI radioiodine ablation, PPT post-partum thyroiditis

### Post-ramadan outcomes: post-ramadan TSH, thyroid status, preference, and adherence

**Fig. 2:**
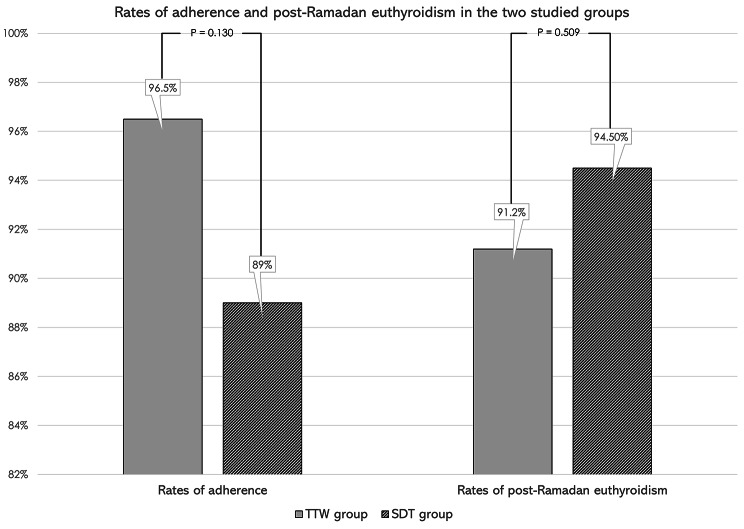
Rates of adherence and post-Ramadan euthyroidism in the two studied groups. TTW: Twice or thrice weekly levothyroxine group, SDT: Standard daily therapy group

Most patients in both groups were still euthyroid post-Ramadan, 91.2% of TTW group versus 94.5% of SDT group, without significant difference in between groups [P = 0.509] (Fig. 2). Post-Ramadan TSH remained significantly higher in TTW group compared to SDT group, mean TSH was 2.30 ± 1.25 µIU/L for TTW group versus 1.69 ± 1.70 µIU/L for SDT group [P < 0.001] (Table 1).

Fasting Ramadan had a different impact in both studied groups regarding post-Ramadan TSH relative to pre-Ramadan TSH. In TTW group, post-Ramadan TSH increased significantly to 2.30 ± 1.25 µIU/L versus 1.80 ± 0.88 µIU/L pre-Ramadan [P = 0.014], while in SDT group, post-Ramadan TSH increased, but non significantly to 1.69 ± 1.70 µIU/L versus 1.39 ± 0.72 µIU/L pre-Ramadan [P = 0.209] (Table 2).

**Table 2:** Comparison between Pre-Ramadan and Post-Ramadan TSH in each group

TSH	Pre-Ramadan	Post-Ramadan	Z	p
**TTW group**				
Mean ± SD.	1.80 ± 0.88	2.30 ± 1.25	2.459^*^	0.014^*^
Median (Min. – Max.)	1.81 (0.30–3.90)	2.08 (0.08–7.50)		
**SDT group**				
Mean ± SD.	1.39 ± 0.72	1.69 ± 1.70	1.257	0.209
Median (Min. – Max.)	1.17 (0.30–3.68)	1.52 (0.08–15.1)		

SD: **Standard deviation** Z: **Wilcoxon signed ranks test**

p: p value for comparing between **Pre-Ramadan** and **Post-Ramadan**

*: significant at p value < 0.05

TTW Twice or thrice weekly, SDT Standard daily therapy

In TTW group: L-T4 regimen preferences were 64.9%, 29.8%, 5.3%, and 0% for regimens 1, 2, 3, and 4 respectively. In SDT group: L-T4 regimen preferences were 35.2%, 45.1%, 7.7%, and 12% for regimens 1, 2, 3, and 4, respectively. TTW group preferred regimen 1 (64.9%) significantly more than SDT group (35.2%) [P = 0.001] (Table 1).

Adherence to L-T4 regimens was higher in TTW group at 96.5% versus 89% in SDT group, however, without statistical significance [P = 0.13] (Fig. 2).

There was no relation between adherence to, nor preference of L-T4 regimens and post-Ramadan TSH in both groups. Pre-Ramadan TSH correlated significantly to post-Ramadan TSH only in SDT group (Spearman coefficient 0.314 [P = 0.002]).

## Discussion

TTW levothyroxine showed high rates of adherence to levothyroxine therapy during Ramdan and high rates of post-Ramadan euthyroidism that were similar to SDT levothyroxine with better convenience and more involvement in the family gatherings that usually take place at Ramadan. The present study confirms the safety and efficacy of TTW L-T4 demonstrated in the previously published pilot study one year ago [7].

Several studies reporting the impact of fasting Ramadan on thyroid function in hypothyroid patients have included exclusively patients rendered euthyroid on stable L-T4 therapy. Post-Ramadan dysthyroidism was found to occur at variable rates. Oudghiri et al. reported 19%, Elsherbiny reported 20%, Alkaf et al. reported 25.4%, Elsherbiny reported 25.7%, and El-Kaissi et al. reported 32% of their patients being dysthyroid in post-Ramadan visit [6, 8, 10–12].

In the previously published pilot study, post-Ramadan dysthyroidism was 18.2% in the twice or thrice weekly L-T4 group versus 17.7% in the daily L-T4 group, without statistical difference [7]. In the present study, post-Ramadan dysthyroidism was only 8.8% in the twice or thrice weekly L-T4 group versus 5.5% in the daily L-T4 group, also without statistical difference.

Pre-Ramadan TSH, age, gender, and L-T4 regimen were among the determinants of post-Ramadan thyroid status, however, adherence was the most consistently reported determinant [6, 8, 10–12].

Nonadherence to L-T4 may be due to pure forgetfulness, confusion about its permanency, drug intolerance or merely fear of adverse effects, or an absence of symptoms [13, 14]. However, the need to take on a daily basis, on an empty stomach an hour before food is definitely burdensome [15]. This is specifically true in Ramadan fasting when patients need either to extend their fasting hours by one more hour if they take L-T4 before Iftar or break their fast and then have a second shorter fast for 3–4 h to empty their stomach to take L-T4 before Suhor [8]. This is further complicated by the frequent family gatherings at Iftar meal when patients are socially obliged to share the meal with the family, limiting their ability to take L-T4 at Iftar.

This has been reflected in research with high rates of reported nonadherence to L-T4 during Ramadan. El-Kaissi et al. reported 30%, Oudghiri et al. reported 36%, Dabbous et al. reported 64%, and Karoli et al. reported 75% of their patients being nonadherent to LT4 instructions during Ramadan [10, 16–18]. In the present study, 96% of the patients in the twice or thrice weekly L-T4 group were adherent to L-T4 instructions compared to 89% in the standard daily L-T4 group, although without statistical significance. In the previously published pilot study, adherence to L-T4 instructions also exceeded 90% at 91% in the twice or thrice weekly L-T4 group versus 88.5% in the daily L-T4 group, also without statistical difference [7].

The use of once weekly levothyroxine is supported by the fact that it has a long elimination half-life of 7 days, and an autoregulatory mechanism exists carried out by deiodinases to prevent hyper- and hypofunction at the beginning and the end of the dosing interval, respectively [19]. The efficacy and safety of once weekly L-T4 have been confirmed in 3 randomized crossover studies [15, 19, 20]. Although once weekly L-T4 is not used routinely in hypothyroid patients, it has shown higher efficacy in achieving better control in patients with treatment refractory hypothyroidism [3, 14].

Based on the same principles, twice weekly and every other day L-T4 are alternatives to standard daily L-T4 that proved to be as safe and effective as SDT [4, 5]. The need for a slightly higher L-T4 dose when switching a patient from daily to once weekly L-T4 dosing was suggested by Grebe et al. to achieve complete euthyroid state, however this was never tested in a subsequent research. [19] Rajput et al. and Bornschein et al. didn’t make a similar suggestion and considered the same daily L-T4 dose given as a once weekly dose an equal alternative. [15, 20] Taylor et al. and Dayal et al. also didn’t recommend a higher L-T4 dose when using twice weekly and alternate day L-T4 dosing. [21] This unsettled question about the need to increase the L-T4 dose when switching to weekly L-T4 dosing may also explain the significantly increased TSH in TTW versus SDT groups in the present study.

In the present study, twice or thrice weekly L-T4 aimed to decrease the burden of altered routine for hypothyroid patients fasting during Ramadan from a daily routine to only 2 or 3 days, thus allowing 4–5 days a week of usual routine for any Muslim fasting Ramadan. Instead of once weekly mega L-T4 dose, the weekly dose was divided into 2 or 3 weekly successive doses to be more acceptable and less worrisome for patients. The choice of dosing days to be Saturdays and Sundays ± Mondays were specifically chosen to avoid usual days of family gatherings; these are Thursdays and Fridays.

The main limitation of the present study is the long list of exclusions – cardiac diseases, pregnancy, and history of thyroid cancer – making TTW L-T4 not applicable for many hypothyroid patients. Another limitation of the present study includes significantly higher pre-Ramadan TSH in the TTW group which may have resulted in lower (yet non-significant) rates of post Ramadan euthyroidism. A third limitation is the significantly younger age in the TTW group, unlike a previous study linking younger age to decreased adherence to L-T4 [6], in the present study the TTW group showed higher adherence rates during Ramadan (yet non-significant), however, pre-Ramadan adherence was not assessed. The strengths of the present study include the novel utility of twice or thrice weekly L-T4 dosing during Ramadan resulting in a high rate of adherence (96.5%), a high rate of post-Ramadan euthyroidism (91.2%), and better convenience and potentially better quality of life. A final point of strength is the larger number of participants relative to the previous pilot study.

## Conclusions

In conclusion, twice or thrice weekly L-T4 during Ramadan had similarly high rates of adherence (96.5%) and post Ramadan euthyroidism (91.2%) to standard daily L-T4. Twice or thrice weekly L-T4 offers more convenience to fasting hypothyroid patients, permitting more active involvement in Ramadan social life.

## Data Availability

The datasets generated during and/or analysed during the current study are available from the corresponding author on reasonable request.

## Abbreviations

L-T4: Levothyroxine
TTW: Twice or thrice weekly
SDT: Standard daily therapy
ECLIA: Electrochemiluminescence immunoassay
ETA: European thyroid association
